# Supplementary material for: Discovery and application of insertion-deletion (INDEL) polymorphisms for QTL mapping of early life-history traits in Atlantic salmon
Source: BMC Genomics. 2010 Mar 8;11:156. doi: 10.1186/1471-2164-11-156 (PMC2838853; doi:10.1186/1471-2164-11-156)
Supplement: Additional file 2 — Information on developed 76 locus single-run INDEL panel in Atlantic salmon. Information on fluorescence labeling, primer concentrations, PCR pooling and links to alignments, INDEL motifs and GENESCAN (Burge and Karlin 1997) predictions of genes/exons are available in html format. [file 1471-2164-11-156-S2.ZIP › Additionalfile2/snpsummary3838.html]

```
Cluster 846 Contig 1

prev  Summary    Contig List  next
```

Size of Consensus sequence = 1796

Number of sequences = 24

Minimum redundancy = 6

Key

A gi|117464028|gb|EG796247.1|EG796247 EST\_ssal\_evd\_15942 ssalevd thymus Salmo salar cDNA Salmo salar cDNA clone ssal\_evd\_520\_107\_fwd 3', mRNA sequence  
B gi|117837541|gb|EG910237.1|EG910237 EST\_ssal\_evf\_12371 ssalevf mixed\_tissue Salmo salar cDNA Salmo salar cDNA clone ssal\_evf\_515\_009\_rev 5', mRNA sequence  
C gi|117837540|gb|EG910236.1|EG910236 EST\_ssal\_evf\_12370 ssalevf mixed\_tissue Salmo salar cDNA Salmo salar cDNA clone ssal\_evf\_515\_009\_fwd 3', mRNA sequence  
D gi|59834475|gb|DN140158.1|DN140158 SGP266180 Atlantic salmon Head kidney cDNA library Salmo salar cDNA clone FN4-3508 5', mRNA sequence  
E gi|117845603|gb|EG918299.1|EG918299 EST\_ssal\_evf\_57366 ssalevf mixed\_tissue Salmo salar cDNA Salmo salar cDNA clone ssal\_evf\_576\_361\_rev 5', mRNA sequence  
F gi|117494671|gb|EG826888.1|EG826888 EST\_ssal\_eve\_39491 ssaleve thyroid Salmo salar cDNA Salmo salar cDNA clone ssal\_eve\_553\_285\_rev 5', mRNA sequence  
G gi|117494672|gb|EG826889.1|EG826889 EST\_ssal\_eve\_39492 ssaleve thyroid Salmo salar cDNA Salmo salar cDNA clone ssal\_eve\_553\_285\_fwd 3', mRNA sequence  
H gi|117536314|gb|EG867759.1|EG867759 EST\_ssal\_eve\_29175 ssaleve thyroid Salmo salar cDNA Salmo salar cDNA clone ssal\_eve\_539\_241\_fwd 3', mRNA sequence  
I gi|117536313|gb|EG867758.1|EG867758 EST\_ssal\_eve\_29174 ssaleve thyroid Salmo salar cDNA Salmo salar cDNA clone ssal\_eve\_539\_241\_rev 5', mRNA sequence  
J gi|117848940|gb|EG921636.1|EG921636 EST\_ssal\_evf\_829 ssalevf mixed\_tissue Salmo salar cDNA Salmo salar cDNA clone ssal\_evf\_005\_083\_rev 5', mRNA sequence  
K gi|24345469|gb|CA044282.1|CA044282 ssaleha002094 gut Salmo salar cDNA, mRNA sequence  
L gi|117531672|gb|EG863117.1|EG863117 EST\_ssal\_eve\_56097 ssaleve thyroid Salmo salar cDNA Salmo salar cDNA clone ssal\_eve\_576\_178\_rev 5', mRNA sequence  
M gi|117521256|gb|EG852983.1|EG852983 EST\_ssal\_eve\_13878 ssaleve thyroid Salmo salar cDNA Salmo salar cDNA clone ssal\_eve\_518\_325\_rev 5', mRNA sequence  
N gi|117548582|gb|EG880027.1|EG880027 EST\_ssal\_eve\_23385 ssaleve thyroid Salmo salar cDNA Salmo salar cDNA clone ssal\_eve\_531\_335\_rev 5', mRNA sequence  
O gi|117847367|gb|EG920063.1|EG920063 EST\_ssal\_evf\_58955 ssalevf mixed\_tissue Salmo salar cDNA Salmo salar cDNA clone ssal\_evf\_579\_086\_rev 5', mRNA sequence  
P gi|117829535|gb|EG902231.1|EG902231 EST\_ssal\_evf\_40909 ssalevf mixed\_tissue Salmo salar cDNA Salmo salar cDNA clone ssal\_evf\_554\_234\_rev 5', mRNA sequence  
Q gi|117521257|gb|EG852984.1|EG852984 EST\_ssal\_eve\_13879 ssaleve thyroid Salmo salar cDNA Salmo salar cDNA clone ssal\_eve\_518\_325\_fwd 3', mRNA sequence  
R gi|117848941|gb|EG921637.1|EG921637 EST\_ssal\_evf\_830 ssalevf mixed\_tissue Salmo salar cDNA Salmo salar cDNA clone ssal\_evf\_005\_083\_fwd 3', mRNA sequence  
S gi|117847366|gb|EG920062.1|EG920062 EST\_ssal\_evf\_58954 ssalevf mixed\_tissue Salmo salar cDNA Salmo salar cDNA clone ssal\_evf\_579\_086\_fwd 3', mRNA sequence  
T gi|117548583|gb|EG880028.1|EG880028 EST\_ssal\_eve\_23386 ssaleve thyroid Salmo salar cDNA Salmo salar cDNA clone ssal\_eve\_531\_335\_fwd 3', mRNA sequence  
U gi|117531673|gb|EG863118.1|EG863118 EST\_ssal\_eve\_56098 ssaleve thyroid Salmo salar cDNA Salmo salar cDNA clone ssal\_eve\_576\_178\_fwd 3', mRNA sequence  
V gi|117829534|gb|EG902230.1|EG902230 EST\_ssal\_evf\_40908 ssalevf mixed\_tissue Salmo salar cDNA Salmo salar cDNA clone ssal\_evf\_554\_234\_fwd 3', mRNA sequence  
W gi|117845604|gb|EG918300.1|EG918300 EST\_ssal\_evf\_57367 ssalevf mixed\_tissue Salmo salar cDNA Salmo salar cDNA clone ssal\_evf\_576\_361\_fwd 3', mRNA sequence  
X gi|89852987|gb|DY709110.1|DY709110 EST\_ssal\_rgb2\_64849 ssalrgb2 mixed\_tissue Salmo salar cDNA Salmo salar cDNA clone ssal\_rgb2\_604\_236\_rev 5', mRNA sequence

2 SNPs detected

A B C D E F G H I J K L M N O P Q R S T U V W X  cosegregation weighted

373 - C C - - C C C C A - - C C C - C . C . . . . .   1/2 37.50
374 - A A - - A A A A A - - A A A - A . A . . . . .   1/2 37.50
